# Sphingosines Derived from Marine Sponge as Potential Multi-Target Drug Related to Disorders in Cancer Development

**DOI:** 10.3390/md13095552

**Published:** 2015-08-25

**Authors:** Renata Biegelmeyer, Rafael Schröder, Douglas F. Rambo, Roger R. Dresch, João L. F. Carraro, Beatriz Mothes, José Cláudio F. Moreira, Mário L. C. da Frota Junior, Amélia T. Henriques

**Affiliations:** 1 Laboratório de Farmacognosia, Faculdade de Farmácia, Universidade Federal do Rio Grande do Sul (UFRGS), Porto Alegre, RS 90610-000, Brazil; E-Mails: douglasrambofar@hotmail.com (D.F.R.); rogdresch@gmail.com (R.R.D.); amelia.henriques@ufrgs.br (A.T.H.); 2 Centro de Estudos em Estresse Oxidativo, Dpto. Bioquímica, Universidade Federal do Rio Grande do Sul (UFRGS), Porto Alegre, RS 90035-000, Brazil; E-Mails: rafaelschroder@terra.com.br (R.S.); jcfm@ufrgs.br (J.C.F.M.); 3 Museu Nacional, Departamento de Invertebrados, Universidade Federal do Rio de Janeiro (UFRJ), Rio de Janeiro, RJ 20940-040, Brazil; E-Mail: joao.porifera@gmail.com; 4 Fundação Zoobotânica, Museu de Ciências Naturais, Porto Alegre, RS 90690-000, Brazil; E-Mail: beatrizmothes@gmail.com

**Keywords:** *H. tubifera*, sphingosines, cytotoxic, anticoagulant, antioxidant

## Abstract

*Haliclona tubifera*, marine sponge species abundant in Brazilian coastline, presents only a few papers published in the literature. Recently, we have reported the isolation of two modified C18 sphingoid bases: (2*R*,3*R*,6*R*,7*Z*)-2-aminooctadec-7-ene-1,3,6-triol and and (2*R*,3*R*,6*R*)-2-aminooctadec-1,3,6-triol. In order to continue our research, in this work aimed at the biological investigation of fractions that led to the isolation of these compounds. We evaluated the cytotoxic effect of marine sponge *H. tubifera* fractions in glioma (U87) and neuroblastoma (SH-SY5Y) human cell lines. In addition, considering the link between cancer, imbalance of reactive oxygen species and coagulation disorders, we also investigated the *in vitro* effects on blood coagulation and their redox properties. We showed that the ethyl acetate (EtOAc) fraction, rich in sphingoid bases, had important cytotoxic effects in both cancer cell lines with an IC_50_ < 15 μg/mL and also can inhibit the production of peroxyl radicals. Interestingly, this fraction increased the recalcification time of human blood, showing anticoagulant properties. The present study indicates the sphingosines fraction as a promising source of chemical prototypes, especially multifunctional drugs in cancer therapy.

## 1. Introduction

Cancer is considered a leading cause of death worldwide and estimates reports that, in 2030, 27 million incident cases of cancer and 17 million of deaths are expected [1]. Among several kinds of cancers, brain tumors are a serious and life-threatening condition, mainly because they can destroy and compress normal brain tissue, causing several types of damage [2]. In addition, the severity of this kind of tumor has been associated with metastatic processes [3]. Gliomas are considered the most devastating primary tumors in the brain [4], while neuroblastoma is the most common extracranial solid cancer in childhood [5].

A strong link between cancer and hypercoagulation has been reported. Approximately 50 percent of patients with a solid tumor have thromboses. Moreover, 95 percent of patients with cancer showed activation in the coagulation system [6,7]. These tumors have been linked with increased tissue factor (TF), which is the main physiological activating player of the blood clotting extrinsic system. TF is produced as part of an inflammatory response, activated by the presence of a tumor, considered a foreign organism [8].

Reactive oxygen species (ROS) and oxidative stress play an important role in the etiology and progression of pathological processes. A leading cause of cancer is associated with increased intrinsic ROS stress, due to mitochondrial dysfunction, oncogenic stimulation and increased metabolic activity [9,10]. Moreover, oxidative stress affects circulating proteins and is associated with an abnormal coagulative pattern [11].

**Figure 1:**
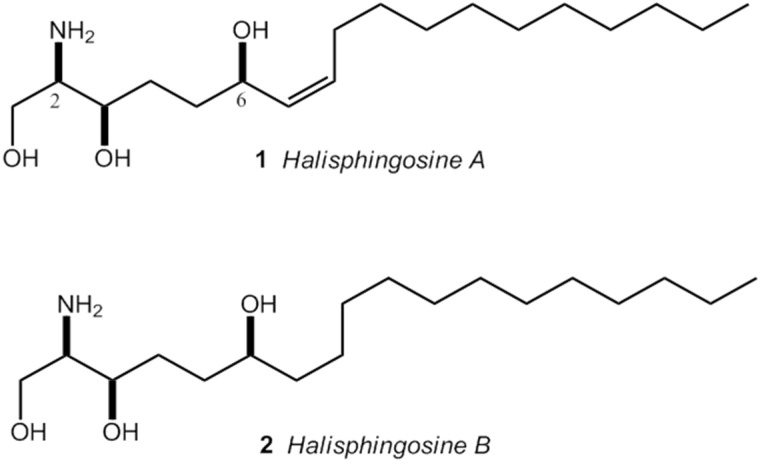
Sphingoid bases isolated from EtOAc fraction of *H. tubifera*: (2*R*,3*R*,6*R*,7*Z*)-2-aminooctadec-7-ene-1,3,6-triol (**1**); and (2*R*,3*R*,6*R*)-2-aminooctadec-1,3,6-triol (**2**) [14].

Despite the variety of drugs for cancer treatment, issues around therapeutic selectivity of antineoplastics and phenotype of resistance to multiple drugs give support to the search for new molecules with antiproliferative properties and, most importantly, new therapeutic modalities [10]. In this context, the marine environment, and especially marine sponges, appeared as a potential source of biologically active compounds [12]. Among marine sponges, the genus *Haliclona* has been widely studied, with approximately 150 papers indexed on Pubmed. On the other hand, *Haliclona tubifera*, a species abundant in Brazilian coastline, presents only a few papers published in the literature, by our research group. The first one, about biological properties in crude extracts [13]. Recently, we have reported the isolation of two modified long-chain sphingoid bases, named Halisphingosines A and B (Figure 1), and the absolute configuration of these compounds was also assigned [14].

In order to continue our study with the marine sponge *Haliclona tubifera*, this research aimed at the biological investigation of fractions that led to the isolation of sphingoid bases. In this sense, we evaluated the cytotoxic effect of fractions of *H. tubifera* in U87 human glioma and SH-SY5Y human neuroblastoma cell lines. Moreover, considering the link between cancer, imbalance of reactive oxygen species and coagulation disorders, we also investigated the *in vitro* effects on blood coagulation and their redox properties.

## 2. Results and Discussion

### 2.1. Cytotoxic Evaluation

The cytotoxic results of *H. tubifera* fractions in U87 human glioma and SH-SY5Y human neuroblastoma cell lines are shown in Table 1. We can observe that the fractionation allows find the main active fraction, since the EtOAc fraction showed a significant drop in cancer cell lines viability while hexane and aqueous fractions did not produce any cytotoxic effect after treatment for 24 h. EtOAc fraction trigged a cytotoxic effect slightly higher for glioma cells, with an IC_50_ = 12.47 μg/mL, compared with the results on neuroblastoma cells that had an IC_50_ around 17 μg/mL.

**Table 1:** *In vitro* growth inhibitory activity against human glioma and neuroblastoma cell lines of *H. tubifera* fractions.

*H. tubifera* Fractions	IC_50_ (μg/mL) ^a^	
	U87	SH-SY5Y
Ethyl Acetate	12.47 ± 1.28	16.72 ± 1.24
Aqueous	na	na
Hexane	na	na

^a^ All values given are the mean ± SEM. na: not active.

The potential biological effects of the marine sponge *H. tubifera* were first reported by Monks and coworkers, which reported the cytotoxicity of the organic extract against colorectal cancer (HT-29), lung cancer (NCI-H460) and another glioma tumor cell line (U-373), with an IC_50_ of about 30 μg/mL [13]. Our results demonstrated a cytotoxic effect using others cancer cell lines and a reduction in IC_50_, after fractionation of the extracts.

The EtOAc fraction of *H. tubifera* is mainly constituted by sphingoid bases through analysis using TLC (ninhydrin bands) and ^1^H NMR spectrum. The major compound was isolated as an *N*-Boc derivative and identified as (2*R*,3*R*,6*R*,7*Z*)-2-aminooctadec-7-ene-1,3,6-triol (Halisphingosine A) [14]. We also characterized another, minor, new C18 sphingoid of *H. tubifera* EtOAc fraction, structurally related to Halisphingosine A (without a double bond in C-7), named Halisphingosine B (Figure 1) [14]. Efforts are being performed to synthesize these compounds to test for biological activities.

Sphingosines are gaining recognition as important signaling mediator of apoptosis, mainly because they are not exclusively confined to membrane fractions, thus, being an ideal second messenger. Possible mechanisms involved in sphingosine-mediated cell death are related with it potential to modulate kinases, mainly: Protein kinase C (PKC), protein kinase A (PKA) and protein kinase Cδ (PKCδ). The activation of PKA and cleaving of PKCδ are associated with the regulation of dimeric 14-3-3 protein function, which displays a vital clue of control pro-apoptotic mediators, such as Bad and signal-regulating kinase 1 (ASK-1). The activation of Jun N-terminal kinases (JNK) is subsequently regulated by ASK-1 [15].

**Figure 2:**
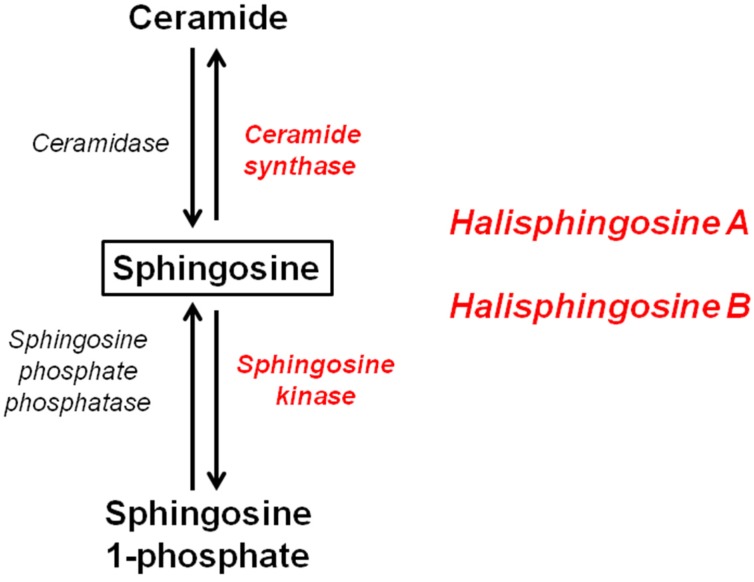
Sphingolipid biosynthetic pathway, showing enzymes (in red) that Halisphingosines A and B could be substrate.

Research studies have shown that the sphingolipid biosynthetic pathway is activated in response to stress conditions, leading to the accumulation of ceramide and sphingosine in apoptotic cells [15]. Considering that Halisphingosines A and B are the major compounds of the EtOAc fraction of *H. tubifera*, these sphingosines could be the substrate of ceramide synthase and sphingosine kinase, as shown in Figure 2. The action of these enzymes can convert sphingosine to ceramide by a scavenger pathway or be converted to sphingosine-1-phosphate (S1P). Ceramide exert antiproliferative effects inducing apoptotic mediators, whereas S1P promotes cell survival and inhibition of apoptosis [16,17,18]. Specifically, for the cell lines tested in our work, Munoz-Sáez and coworkers [19] showed that all doses tested for S1P protects necrosis in neuroblastoma cells (SH-SY5Y). On the other hand, Bernhart and coworkers [20] revealed the importance of sphingolipid synthesis and signaling for attenuating the proliferation of U87MG glioma cells. In this sense, we can infer that the possible cytotoxic mechanism of the EtOAc fraction of *H. tubifera* is associated with an induction of apoptotic mediators and also due to the production of metabolites (ceramides).

### 2.2. Effects on Blood Coagulation

**Figure 3:**
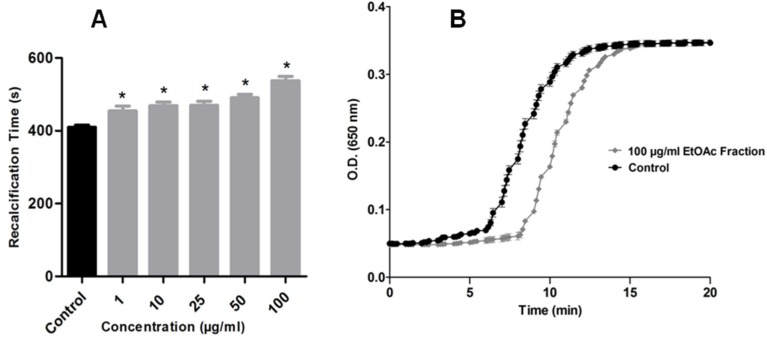
(**A**) Effect of marine sponge *H. tubifera* on blood coagulation: Anticoagulant activity for the EtOAc fraction; (**B**) Representative profile of the highest concentration that exhibited anticoagulant activity; Bars represent mean ± SEM. Asterisk (*) used when *p* < 0.05 compared to control (1-way ANOVA followed by Tukey’s test).

Considering the correlation of cancer and hypercoagulation condition, the effects of *H. tubifera* fractions upon clotting, through calcium-induced clotting time of citrated human plasma was analyzed. We demonstrated that, in addition to antitumor activity, the EtOAc fraction of *H. tubifera* also affected blood coagulation. Figure 3A shows the anticoagulant activity of the EtOAc fraction in a dose-dependent manner. All concentrations tested (1.0–100 μg/mL) changed the clotting time, comparing to the control. In the highest concentration tested, a significantly longer recalcification time was observed (approximately 9 min, Figure 3B), while the control plasma took approximately over 6 min to initiate coagulation.

The hypercoagulation process in patients with cancer is mainly caused by the tumor growing, neo-angiogenesis and dysfunction of affected organs [6]. The marine environment plays an important role in the search for anticoagulant products, and the vast majority of these compounds are polysaccharides [21,22,23]. Nevertheless, only a few anticoagulant products were isolated from marine sponges: A sesquiterpene from *Coscinoderma mathewsi* [24] and a peptide from the Australian sponge *Lamellodysidea chlorea* [25].

### 2.3. Redox Properties

**Figure 4:**
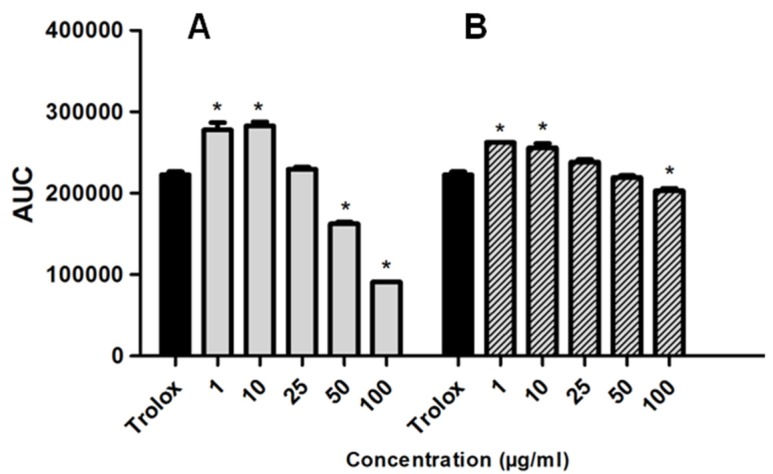
TRAP from marine sponge *H. tubifera*: the EtOAc fraction (**A**); and the aqueous fraction (**B**) The effect of different concentrations of fractions on free radical induced chemiluminescence (CL) was measured as AUC. Trolox (0.05 μg/mL) and was used as a standard antioxidant. Bars represent mean ± SEM. Asterisk (*) used when *p* < 0.05 compared to control (1-way ANOVA followed by Tukey’s test).

**Figure 5:**
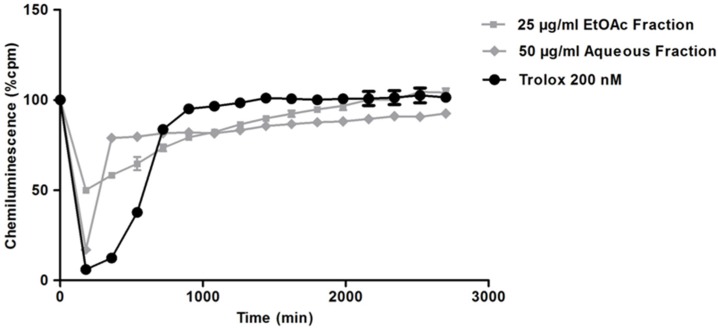
CL intensity (% cpm) measured after the addition of *H. tubifera* fractions. The CL profile of samples in concentration that exhibited similar AUC with the standard Trolox is represented.

The redox properties of the marine sponge *H. tubifera* fractions were evaluated based on the capacity of samples to scavenge peroxyl radicals, generated by thermal decomposition of AAPH and compared with the antioxidant standard Trolox (water-soluble vitamin E analog). Significant dose-dependent antioxidant effects of EtOAc and aqueous fractions are shown in Figure 4. The EtOAc fraction at 25 μg/mL had an AUC similar to that of Trolox; however, could not inhibit the production of free radicals at initial time to the same extent as Trolox (Figure 5). The aqueous fraction (50 μg/mL), besides inhibiting free radicals, such as Trolox, sustains this effect for longer periods (Figure 5). The Trolox equivalent antioxidant capacity (TEAC) allowing to comparing antioxidant activity of fractions and results, are summarized in Table 2. Both fractions presented a similar TEAC value, though the EtOAc fraction was slightly more potent to scavenge free radicals than the aqueous fraction.

**Table 2:** Antioxidant effect expressed as Trolox equivalent antioxidant capacity (TEAC) of *H. tubifera* fractions.

*H. tubifera* Fractions	TEAC (μM Trolox/g Marine Sponge)
Ethyl Acetate	8.0
Aqueous	5.22
Hexane	na

na: not active.

The visualization of ninhydrin positive bands as for EtOAc fraction was not possible through the analysis of TLC for aqueous fraction. Thus, we can infer that the inhibition of free radicals by aqueous fractions should be produced by other compounds but not by sphingoid bases. The negative results for cytotoxic and anticoagulant assays also corroborate that the chemistry composition of the aqueous fraction do not present Halisphingosines A and B.

Moreover, oxidative stress may promote an abnormal coagulative pattern and alter the structure and function of coagulative proteins [26], and play an important part in the initiation and progression of cancer [27]. Among the tested fractions, the EtOAc fraction also showed highest activity to scavenge peroxyl radicals, in a chemioluminescence assay. Several *in vitro*, *in vivo*, and epidemiological studies have demonstrated that antioxidants exert cancer-preventive potential [28]. Strategies that modulate the cellular redox potential are being applied to kill cancer cells [29] and antioxidants may be able to slow down or inhibit the progression of premalignant lesions to cancer [30].

## 3. Experimental Section

### 3.1. Materials

MTT (3-(4,5-dimethyl)-2,5-diphenyl tetrazolium bromide), Hepes, Trolox and Warfarin were purchased from Sigma-Aldrich (St. Louis, MO, USA). Methanol, Ethyl Acetate and Hexane (high-performance liquid chromatography (HPLC) grade) were obtained from Tedia (Fairfield, CA, USA).

### 3.2. Sponge Collection

Samples of marine sponge *Haliclona tubifera* were collected manually at depths of between 10 and 15 m, in locations along the coastline of the State of Santa Catarina, Brazil. A specimen of the sponge was deposited at the Museu de Ciências Naturais, Porifera collection, of the Fundação Zoobotânica do Rio Grande do Sul, Brazil.

### 3.3. Preparation of Extracts and Fractions

The marine sponge samples were previously freeze-dried and, then, extracts were prepared using an Ultra-Turrax system (Marconi, Piracicaba, SP, Brazil) with methanol. The raw methanol extracts were partitioned with other solvents to obtain the initial aqueous, hexane and ethyl acetate fractions. Firstly, the volume of the methanol extract was reduced. Then, 10% water was added. The methanol-aqueous suspension was partitioned against hexane. Then, methanol was evaporated using a rotary evaporator (Büchi Labortechnik AG, Flawil, Switzerland). The remaining aqueous suspension was partitioned against ethyl acetate.

### 3.4. Cell Cultures

The human glioma (U87) and human neuroblastoma (SHSY5Y) cell lines were obtained from American Type Culture Collection (Rockville, MD, USA). Glioma cells were grown and maintained in low glucose Dulbecco’s modified Eagle’s medium (DMEM; Gibco BRL, Carlsbad, CA, USA), containing 0.1% fungizone, 100 U/L gentamicin and supplemented with 10% fetal bovine serum, and neuroblastoma cells in a mixture 1:1 of Ham’s F12 and Dulbecco Modified Eagle Medium (DMEM) supplemented with 10% heat-inactivated FBS, 2 mM of glutamine, 0.28 μg/μL of gentamicin and 250 μg of amphotericin B. Cells were plated in 96-well plates (10^4^/well) and kept at 37 °C in a humidified atmosphere with 5% CO_2_. Every 3 days, cell media was replaced and all treatments were performed when cell confluence reached 70%–80%.

### 3.5. Treatments

Aqueous and organic fractions were dissolved in water and dimethylsulfoxide (DMSO), to final concentrations of 10 mg/mL and 50 mg/mL (*w*/*v*), respectively. Successive dilutions were made with DMEM medium to final concentrations, ranging from 1.0 to 100 μg/mL. The cultures were treated with sponge extracts and fractions for 24 h. DMSO (0.25% final concentration) was proven not to affect the experiments. Control cultures were performed identically, but without extracts.

### 3.6. Assessment of Glioma and Neuroblastoma Cell Viability

Cell viability was performed by the quantification of the 3-(4,5 dimethylthiazole-2-yl)-2,5-diphenyltetrazolium bromide (MTT) reduction to a blue formazan product by viable cells. After treatments, the medium was discarded and a new medium containing 0.5 mg/mL MTT was added. The cells were incubated for 45 min at 37 °C in a humidified atmosphere with 5% CO_2_. At the end of the incubation, this medium was removed and DMSO was added to solubilize formazan crystals for 30 min. Absorbance was measured at 550 nm (test) and 690 nm (reference) in a SoftMax Pro Microplate Reader (Molecular Devices^®^, Sunnyvale, CA, USA). The half maximal inhibitory concentration values (IC_50_) were estimated from a semilog plot of fractions and extract concentrations *versus* percentage of inhibition of tumor cell lines growth.

### 3.7. Evaluation of Antioxidant Activity Using the Total Reactive Antioxidant Potential Method

The total reactive antioxidant potential (TRAP) is extensively employed to verify the antioxidant capacity of samples *in vitro*. This method is based on the quenching of luminol-enhanced chemiluminescence (CL) derived from the thermolysis of 2,20-azo-bis (2 amidinopropane)dihydrochloride (AAPH) as the free radical source [31]. The stock solution was prepared with AAPH (10 mM). Luminol (8 nM) in a glycine buffer (0.1 M; pH 8.6) was added. The system was stabilized under constant light intensity (2 h) before the first reading. After, 20 μL of each sample, Trolox or system (glycine buffer) were placed in a 96 cell-plate and the stock solution was adjusted to a final concentration of 200 μL. The CL emitted by the free radical reaction was quantified in a liquid scintillator counter (Wallac 1409, Perkin Elmer, Boston, MA, USA, with 10 s defined as a count time, for 3000 s). The samples (concentration of 50 mg/mL) were adjusted with glycine buffer to reach the final concentrations (1, 10, 25, 50 and 100 μg/mL). The final percentage of DMSO (0.25%) was proven to not change the chemiluminescence (CL) in the system. Trolox was prepared with glycine buffer. The results were expressed as plotting percentage of counts per minute (% cpm) *versus* time (s) and area under curve (AUC). Using the standard curve, which was obtained by plotting the concentration of Trolox and the AUC (between 0.05 and 0.4 μM Trolox), Trolox equivalent antioxidant capacity (TEAC) was estimated.

### 3.8. Clotting Assay

Recalcification time (RT) was assessed using a SpectraMax microplate ELISA reader (Molecular Devices^®^, Sunnyvale, CA, USA) as described by Ribeiro and coworkers [32]. The procedure allows to follow clot formation and to use kinetic parameters for the coagulation process. Previously, 50 μL of human citrated platelet-poor plasma was incubated for 5 min, with 80 μL of 20 mM HEPES, pH 7.4, with or without (control) varied amounts of fractions (1, 10, 25, 50 and 100 μg/mL). Coagulation was triggered by adding CaCl_2_ to a final concentration of 10 mM, and clot formation was analyzed at 37 °C in the SpectraMax system at 650 nm taken at 15-s intervals, for 20 min. The final percentage of DMSO (0.25%) was proven not to affect blood coagulation. Clotting is indicated as a fast and sharp increase in the absorbance after a lag phase. A variation of 0.05 in the absorbance value (onset time) was defined as a measure of recalcification time, using the module included in the instrument’s software.

### 3.9. Statistical Analysis

The *in vitro* experiments were carried out with *n* = 3, while cell culture experiments were conducted with *n* = 6 in two independent experiments. Data were expressed as mean ± standard error of the mean (SEM). The results were evaluated by one-way analysis of variance (ANOVA) followed by Tukey’s Post Hoc Test.

## 4. Conclusions

In summary, this work sheds new light on the study of the marine sponge *Haliclona tubifera*, an abundant species from the southern Brazilian coast. We screened fractions obtained from the crude extract of *H. tubifera* along with their effects on human cancer cell lines, free radical and on blood coagulation in order to identify the main active fraction. The results demonstrate a potential cytotoxic activity in human glioma and neuroblastoma cell lines and also a strong antioxidant capacity of the sphingosines fraction. Moreover, our findings showed that this fraction exerts a significant anticoagulant effect using human citrated plasma samples. In addition, the relationship between the cytotoxic and anticoagulant activity of the sphingosines fraction of *H. tubifera* opens up perspectives for the research of new therapeutic modalities in the cancer therapy. Thus, our findings support to evaluate the biological effects of sphingoid bases previously isolated, and also to keep working on the isolated sphingoid bases from the EtOAc fraction of *H. tubifera*.
